# *Aedes aegypti* saliva impairs M1-associated proinflammatory phenotype without promoting or affecting M2 polarization of murine macrophages

**DOI:** 10.1186/s13071-019-3487-7

**Published:** 2019-05-16

**Authors:** Michele S. Barros, Priscila G. Lara, Monique T. Fonseca, Eduardo H. Moretti, Luciano R. Filgueiras, Joilson O. Martins, Margareth L. Capurro, Alexandre A. Steiner, Anderson Sá-Nunes

**Affiliations:** 1 Laboratory of Experimental Immunology, Department of Immunology, Institute of Biomedical Sciences, University of Sao Paulo, Sao Paulo, SP 05508-000 Brazil; 2 Laboratory of Sepsis Neurobiology, Department of Immunology, Institute of Biomedical Sciences, University of Sao Paulo, Sao Paulo, SP 05508-000 Brazil; 3 Laboratory of Immunopharmacology, Department of Immunology, Institute of Biomedical Sciences, University of Sao Paulo, Sao Paulo, SP 05508-000 Brazil; 4 Laboratory of Immunoendocrinology, Department of Clinical and Toxicological Analyses, School of Pharmaceutical Sciences, University of Sao Paulo, Sao Paulo, SP 05508-000 Brazil; 5 Laboratory of Genetically Modified Mosquitoes, Department of Parasitology, Institute of Biomedical Sciences, University of Sao Paulo, Sao Paulo, SP 05508-000 Brazil; 6 National Institute of Science and Technology on Molecular Entomology, National Council for Scientific and Technological Development (INCT-EM/CNPq), Rio de Janeiro, RJ Brazil

**Keywords:** *Aedes aegypti*, Saliva, Macrophages, Inflammation, M1/M2 polarization, microbicidal activity

## Abstract

**Background:**

During the feeding process, the mouthparts of hematophagous mosquitoes break the skin barrier and probe the host tissue to find the blood. The saliva inoculated in this microenvironment modulates host hemostasis, inflammation and adaptive immune responses. However, the mechanisms involved in these biological activities remain poorly understood and few studies explored the potential roles of mosquito saliva on the individual cellular components of the immune system. Here, we report the immunomodulatory activities of *Aedes aegypti* salivary cocktail on murine peritoneal macrophages.

**Results:**

The salivary gland extract (SGE) of *Ae. aegypti* inhibited the production of nitric oxide and inflammatory cytokines such as interleukin-6 (IL-6) and IL-12, as well as the expression of inducible nitric oxide synthase and NF-κB by murine macrophages stimulated by lipopolysaccharide (LPS) plus interferon-γ (IFN-γ). The spare respiratory capacity, the phagocytic and microbicidal activities of these macrophages were also reduced by *Ae. aegypti* SGE. These phenotypic changes are consistent with SGE suppressing the proinflammatory program of M1 macrophages. On the other hand, *Ae. aegypti* SGE did not influence M2-associated markers (urea production, arginase-1 and mannose receptor-1 expression), either in macrophages alternatively activated by IL-4 or in those classically activated by LPS plus IFN-γ. In addition, *Ae. aegypti* SGE did not display any cytokine-binding activity, nor did it affect macrophage viability, thus excluding supposed experimental artifacts.

**Conclusions:**

Given the importance of macrophages in a number of biological processes, our findings help to enlighten how vector saliva modulates vertebrate host immunity.

## Background

When attempting to feed on a vertebrate host, the mouthparts of females *Aedes aegypti* (Linnaeus, 1762) are inserted into the skin, the primary interface between the body and the environment, and “probe” the tissue to find a suitable vessel or a hemorrhagic pool [1]. During the process, mosquito saliva is inoculated in this microenvironment, assisting in the location of blood vessels and counteracting molecules and resident cells responsible for the host’s hemostasis, inflammatory and adaptive immune responses [2, 3]. While the anticoagulant, antiplatelet and vasodilatory activities of *Ae. aegypti* saliva are addressed in much of the scientific literature [4–9], the role of the species’ salivary components on immune cells remains largely unknown.

In addition to its role as a physical barrier, the skin is now recognized as an immunological organ according to the concept introduced by Dr J. W. Streilein [10] and further developed by himself and many others [11–15]. Among the resident immune response-associated cells in the skin are keratinocytes, mast cells, T lymphocytes (α/β and γ/δ T cells), innate lymphoid cells, dendritic cells, and macrophages. Given the limitation to isolate these cells directly from the skin or study them *in situ* under real life situations, *in vitro* models represent valuable tools and have been extensively employed to evaluate the effects of *Ae. aegypti* salivary components on the phenotype and functions of these cells. Thus, the production of cytokines by keratinocytes [16], dendritic cells [17] and mast cells [18] in response to inflammatory or infectious stimuli was impaired in the presence of *Ae. aegypti* salivary gland extract (SGE). Likewise, some reports showed a decrease in the polyclonal and antigen-specific proliferation of T cells in the presence of the mosquito’s SGE [19–22] and this effect was due to the induction of caspase-3 and caspase-8-dependent cell death [20].

Macrophages play an important role in the onset, maintenance and resolution of inflammatory responses. As one of the major resident cell type in skin [11–15, 23], macrophages also participate in the arthropod vector-vertebrate host interactions, being probably among the first cells exposed to the saliva released during the blood-feeding. Given the restricted information on the role of mosquito salivary components directly on these cells, a more detailed study focused in the activity of *Ae. aegypti* SGE on several parameters of macrophage function is strongly needed. To date, only two studies explored the activity of *Ae. aegypti* salivary components on these cells, both in murine peritoneal macrophages [17, 24]. Macrophages from C3H/HeJ mice infected with West Nile virus or Sindbis virus expressed decreased levels of mRNA to interferon-β (IFN-β) and inducible nitric oxide synthase (iNOS) in the presence of *Ae. aegypti* SGE. In the absence of infection, *Ae. aegypti* SGE reduced the basal levels of interleukin (IL)-12 and increased IL-10 mRNA expression in these cells [17]. Macrophages from C57BL/6 mice, incubated with synthetic cecropins identified in the *Ae. aegypti* genome, decreased the production of nitric oxide (NO) and inflammatory cytokines, and inhibited the expression of iNOS, mitogen-activated protein kinases (MAPKs) and nuclear factor-κB (NF-κB) in lipopolysaccharide (LPS) stimulated macrophages [24]. However, data on the effect of *Ae. aegypti* salivary components in other aspects of macrophage biology are still lacking. Here, we addressed some of these missing aspects by evaluating the role of *Ae. aegypti* SGE on parameters associated with macrophage polarization to the M1 and M2 profiles.

## Methods

### Mice

Female C57BL/6 mice, 6–10-week-old, were bred and maintained at the Isogenic Breeding Unit of the Department of Immunology, Institute of Biomedical Sciences, University of Sao Paulo, Brazil. During all manipulation procedures, animals were maintained under specific pathogen-free conditions and kept under controlled temperature and luminosity, with food and water *ad libitum*.

### Preparation of *Ae. aegypti* SGE

*Aedes aegypti* mosquitoes (male and female) were reared in an insectary at the Department of Parasitology, Institute of Biomedical Sciences, University of Sao Paulo, Brazil where they were fed and mated as previously described [25]. Five- to eight-day-old female adult mosquitoes were used as a source of salivary glands to prepare the SGE as described [20].

### Macrophage isolation and M1/M2 polarization

Mice were intraperitoneally injected with 1 ml of 4% sterile thioglycolate medium (Becton, Dickinson and Company, Sparks, MD, USA). After 4 days, the animals were euthanized and the peritoneal cavity lavage was collected with 3 ml of cold sterile phosphate-buffered saline (PBS). After centrifugation (300×*g* for 5 min at 4 °C), the cell-free supernatant was discarded, the cell pellet was suspended in RPMI 1640 medium (Gibco Invitrogen, Grand Island, NY, USA), diluted in Turk’s solution (4 mg/l gentian violet in 3% acetic acid), and the number of cells was determined by optical microscopy in a Neubauer’s chamber. A suspension containing 1.5 × 10^6^ cells/ml was prepared in RPMI 1640 medium, distributed into sterile 96-well plates in aliquots of 100 μl/well and incubated for 2 h at 37 °C and 5% CO_2_ for macrophage adhesion. Cell monolayers were carefully washed 3 times with warm PBS (at 30 °C) to remove nonadherent cells, and the adherent cells were incubated once more with complete medium [RPMI 1640 supplemented with 10% heat-inactivated fetal bovine serum (FBS), 2 mM l-glutamine, 100 units/ml penicillin, 100 µg/ml streptomycin, 25 mM HEPES and 2.5 × 10^5^ M 2-mercaptoethanol] and cultured overnight at 37 °C and 5% CO_2_. In the next day, the wells were subjected to a new washing step with warm PBS and the adherent cells (macrophages) were stimulated as it follows.

Macrophages prepared as described above were maintained in complete medium (control group) or preincubated with *Ae. aegypti* SGE (concentrations indicated in each figure) for 1 h. Then, macrophages were polarized either to a M1 profile by activation with 10 ng/ml of ultrapure LPS (InvivoGen, San Diego, CA, USA) plus 10 ng/ml of murine IFN-γ (Sigma-Aldrich, St. Louis, MO, USA) or to a M2 profile by incubation with 20 ng/ml of murine IL-4 (Sigma-Aldrich). As suggested by the “Macrophage Activation and Polarization: Nomenclature and Experimental Guidelines”, these cells will be often referred as M(LPS+IFN-γ) or M(IL-4) for M1 and M2, respectively [26].

### Spleen cells

Following euthanasia, spleens from naive mice were aseptically removed and transferred into individual tubes containing 5 ml of RPMI 1640 medium. The organ was macerated by pressing the spleens through a 40-μm pore-size cell strainer (BD Falcon, Franklin Lakes, NJ, USA) with the aid of a sterile syringe plunger. Cells were centrifuged at 300×*g* for 5 min at 4 °C, the supernatant was discarded and the red blood cells were lysed by ACK Lysing Buffer (Gibco Invitrogen). After further washings, the cells were resuspended in complete medium, diluted in Turk’s solution, counted in a Neubauer’s chamber and used as described below.

### Nitric oxide (NO) and cytokine determination

Macrophage cultures were prepared and polarized to M1 or M2 profile as described above. Cell-free supernatant was collected after 48 h and nitrite (NO_2_^−^), a stable and product of NO oxidation, was evaluated in the culture supernatant by Griess reaction as previously described [27, 28]. Briefly, equal volumes of the supernatants and the Griess reagent (1% sulfanilamide in 5% phosphoric acid and 0.1% N-(1-Naphthyl) ethylenediamine dihydrochloride, v/v) were mixed and incubated for 10 min at room temperature. The optical density of each well was evaluated at 554 nm in a spectrophotometer (SpectraMax M3, Molecular Devices, San Jose, CA, USA) and NO_2_^−^ concentrations were deduced from a standard curve prepared with sodium nitrite (NaNO_2_) concentrations dissolved in complete medium.

Macrophage cultures were prepared and polarized to a M1 profile as described above. Cell-free supernatant was collected either after 6 h [for quantification of tumor necrosis factor-α (TNF-α)] or after 48 h (for quantification of IL-6, IL-10 and IL-12). The levels of the cytokines IL-10, IL-12 (p70) and TNF-α in the culture supernatants were assayed by BD OptEIA™ ELISA sets (BD Biosciences, San Diego, CA, USA) and the levels of IL-6 were evaluated by DuoSet ELISA (R&D Systems, Minneapolis, MN, USA), according to the manufacturers’ recommendations. Values were expressed as pg/ml deduced from standard curves of recombinant cytokines ran in parallel. The detection limit for each cytokine analyzed was: 15.6 pg/ml (IL-6 and TNF-α); 31.3 pg/ml (IL-10); and 62.5 pg/ml (IL-12).

### Assessment of cell viability

Peritoneal macrophages and total spleen cells were prepared as described above and maintained in complete medium (control group) or preincubated with different concentrations of *Ae. aegypti* SGE (final concentration: 1 to 40 μg/ml) for 1 h. Macrophages were stimulated with LPS plus IFN-γ (final concentration: 10 ng/ml each) and lymphocytes (used as a control) were stimulated with 0.5 μg/ml concanavalin A (Con A, Sigma-Aldrich). Concomitantly, 25 µl of 0.01% resazurin (prepared in complete medium) were added to all wells. Cell viability was evaluated after 48 h of culture by reading the culture absorbance at 570 and 600 nm in a plate reader and the results are expressed as the difference between those readings as described [20, 29, 30].

Peritoneal macrophages and total spleen cells were prepared as described above and maintained in complete medium (control group) or incubated with different concentrations of *Ae. aegypti* SGE for 4 h. Then, cells were transferred to polypropylene tubes (12 × 75 mm) and centrifuged at 300×*g* for 5 min at 4 °C. After discarding the supernatant, macrophage samples were stained with fluorescence-conjugated anti-F4/80 (BioLegend, San Diego, CA, USA) and anti-CD11b (BD Biosciences) and lymphocyte samples were stained with fluorescence-conjugated anti-CD3 (BioLegend) and anti-CD19 (BD Biosciences) diluted in flow cytometry buffer (PBS containing 1% FBS) for 30 min at 4 °C in the dark. Cells were then washed twice with annexin buffer (10 mM HEPES, 140 mM NaCl, 0.25 mM CaCl) and centrifuged 300×*g* for 5 min at 4 °C. The cell pellet was resuspended in 100 μl of annexin buffer and 5 μl of annexin V-FITC (BioLegend) were added to each sample, which was then incubated in the dark for 10 min at room temperature. Cells were immediately acquired by a FACSCanto II flow cytometer (BD Biosciences) to evaluate the percentage of annexin V^+^ cells in each population. Data was analyzed using the FlowJo software, version 10.0.5 (Tree Star Inc., Ashland, OR, USA).

### Real-time cell metabolism assay

In another set of experiments, the real-time analysis of mitochondrial oxygen consumption rate (OCR) was evaluated by a Seahorse XFe24 Extracellular Flux Analyzer (Agilent, Santa Clara, CA, USA). Peritoneal macrophages were prepared as described and seeded in sterile 24-well Seahorse culture plates at a density of 1.5 × 10^5^ cells/well. Nonadherent cells were removed by two cycles of washing (after 2 and 24 h) as described before and adherent cells were maintained in complete medium (control group) or preincubated with *Ae. aegypti* SGE (40 μg/ml) for 16 h. Cells were then washed 3 times, equilibrated in assay medium and placed in the equipment following the manufacturer’s instructions. Basal OCR as well as the response to the sequential addition of oligomycin [1 μg/ml – for ATP synthase (complex V) inhibition], carbonyl cyanide m-chlorophenyl hydrazone (CCCP; 4.5 μM – for maximal respiratory capacity) and rotenone/ antimycin A [1 μM each – for mitochondrial (complex I and III, respectively) inhibition] was recorded.

### Arginase activity

M1 and M2 macrophages were polarized as described above. After 48 h, cells were collected, washed three times with cold PBS and the cell pellet was resuspended in RIPA lysis buffer (150 mM NaCl, 1% NP40, 0.1% SDS, 50 mM Tris; pH 8.0) and subjected to three freeze-thaw cycles at −20 °C and 37 °C, respectively. Then, the samples were centrifuged at 14,000×*g* for 5 min at 4 °C and the supernatant was transferred to a new tube. Arginase activity was evaluated in cell lysates as previously described [31]. Urea production by arginase was determined spectrophotometrically at 550 nm and calculated using a standard curve generated with urea. Aliquots of the cell lysate were used for protein quantification using BCA Protein Assay Kit (Thermo Fisher Scientific, Rockford, IL, USA), according to the manufacturer’s recommendations. A standard curve with known concentrations of urea was prepared and the rate of urea production divided by the protein concentration of each sample was used as an index for arginase activity.

### Western blot assays

M1 and M2 macrophages were polarized as described above. After 24 h, cells were then washed three times with cold PBS and lysed with RIPA buffer supplemented with 1% phosphatase inhibitors (100 mM sodium fluoride and 100 mM sodium orthovanadate) and with 1% protease inhibitor (Sigma-Aldrich). The lysate supernatant was collected after 10 min of incubation on ice, centrifuged at 14,000×*g* for 10 min at 4 °C and the protein concentration was determined using the BCA Protein Assay Kit (Thermo Fisher Scientific) according to the manufacturer’s instructions.

The entire electrophoresis and transfer processes were performed with reagents and equipment from Invitrogen (Carlsbad, CA, USA). Electrophoresis was performed in a Bolt™ system, according to the manufacturer’s instructions. The equivalent of 15 μg proteins from each sample were diluted in Bolt™ Sample Reducing Agent and Bolt™ LDS Sample Buffer, heated to 70 °C for 10 min and separated by electrophoresis in a Bolt™ Bis-Tris Plus 4–12% gel under a constant current of 200 V for 35 min. The separated proteins were transferred to a nitrocellulose membrane using the iBlot® Dry Blotting System and then was blocked with TBS-T buffer (Tris-buffered saline, 0.1% Tween-20) containing 10% FBS for 2 h. Membranes were washed three times with TBS-T (5 min per wash) and incubated overnight at 4 °C with the following rabbit monoclonal antibodies: anti-iNOS (Cell Signaling Technology, Danvers, MA, USA; 1:10,000), anti-phospho-NF-κB p65 (Cell Signaling Technology; 1:1,000), anti-arginase-1 (Cell Signaling Technology; 1:1,000) and anti-mannose receptor-1 (Proteintech, Chicago, IL, USA; 1:1,000). After further washing, the membranes were incubated for 2 h at room temperature with anti-rabbit secondary antibodies (1:3,000) conjugated with horseradish peroxidase (Cell Signaling Technology). Immunoreactive bands were stained using the chemiluminescent ECL Detection Kit (Thermo Fisher Scientific) and visualized in a photodocumentation system (G:BOX, Syngene, Cambridge, UK). Lastly, the membranes were then washed and incubated for 30 min with anti-β-actin conjugated with horseradish peroxidase (1:10,000) (Sigma-Aldrich) and visualized using ECL Detection Kit as described above. The density of the bands was analyzed with DigiDoc1000 software (Alpha Innotech Corporation, San Leandro, CA, USA). The values were normalized by the total of β-actin present in each sample and expressed as arbitrary units.

### Cytokine-binding activity of SGE

#### Competition assay

High-binding flat bottom 96-well plates (Costar, Cambridge, MA, USA) were coated with anti-mouse IL-6 or anti-IL-12 antibody diluted in carbonate buffer (0.1 M, pH 9.6) overnight at 4 °C. Then, the wells were washed, incubated with blocking buffer (10% FBS in PBS) for 1 h at room temperature and washed again. Serial dilutions of IL-6 and IL-12 (3.9–500 pg/ml), previously incubated for 2 h at 37 °C with blocking buffer only or with different concentrations of SGE (1, 5 and 10 µg/ml), were added to the wells and incubated at room temperature for 2 h. After new washing, the bound IL-6 or IL-12 was detected using a solution containing biotinylated anti-mouse IL-6 or IL-12 antibody plus streptavidin-peroxidase for 1 h. Finally, wells were washed again, the chromogenic substrate (TMB Substrate Reagent Set, BD Biosciences) was added and the plate was incubated at room temperature for 30 min in a dark place for developing color. Reaction was stopped by addition of 1M H_3_PO_4_. The optical density of the wells was read at 450 nm in a spectrophotometer (Molecular Devices).

#### Direct binding assay

High-binding flat bottom 96-well plates (Costar, Cambridge, MA, USA) were coated with SGE (10 µg/ml), anti-mouse IL-6 or anti-IL-12 antibody diluted in carbonate buffer (0.1 M, pH 9.6) overnight at 4 °C. After the washing and blocking steps performed as described above, serial dilutions of IL-6 and IL-12 (3.9–500 pg/ml) were added to the wells and incubated at room temperature for 2 h. After new washing, the bound IL-6 or IL-12 was detected as described above. Reaction was stopped by addition of 1M H_3_PO_4_. The optical density of the wells was read at 450 nm in a spectrophotometer (Molecular Devices).

#### *Escherichia coli* phagocytosis and killing

Macrophage cultures were prepared as described above and maintained in complete medium without antibiotics (control group) or in the presence of *Ae. aegypti* SGE (final concentration: 40 μg/ml) for 1 h. In one set of experiments, the cells were stimulated with *E. coli* strain ATCC 25992 at a multiplicity of infection (MOI) of 10 at 37 °C and 5% CO_2_ for 5 h to evaluate bacterial killing. The medium was collected at the end of the experiment, serially diluted, and then plated on McConkey agar. After 24 h of incubation at 37 °C, plates that displayed isolated colonies were subjected to counting for determination of colony-forming units. In another set of experiments, the cells were incubated with green fluorescent *E. coli* from EZCell™ Phagocytosis Assay Kit (BioVision, San Francisco, CA, USA) for 1 h. At the end of the assay, macrophages were recovered from the plate using cold PBS, stained with fluorescence-conjugated anti-F4/80, and acquired on FACSCanto II flow cytometer (BD Biosciences). *E. coli* uptake rate and the median fluorescence intensity (MFI) of macrophages from both groups were analyzed by the FlowJo software (Tree Star Inc.).

### Statistical analysis

Statistical analysis of differences between means of experimental groups was performed using Student’s *t*-test (for comparison of two groups) or analysis of variance (ANOVA) followed by Tukey’s *post-test* (for three or more groups). A value of *P* < 0.05 was considered statistically significant. Data are expressed as the mean ± standard error of the mean (SEM).

## Results

### *Aedes aegypti* SGE inhibits NO production and iNOS expression in classically activated macrophages

**Fig. 1:**
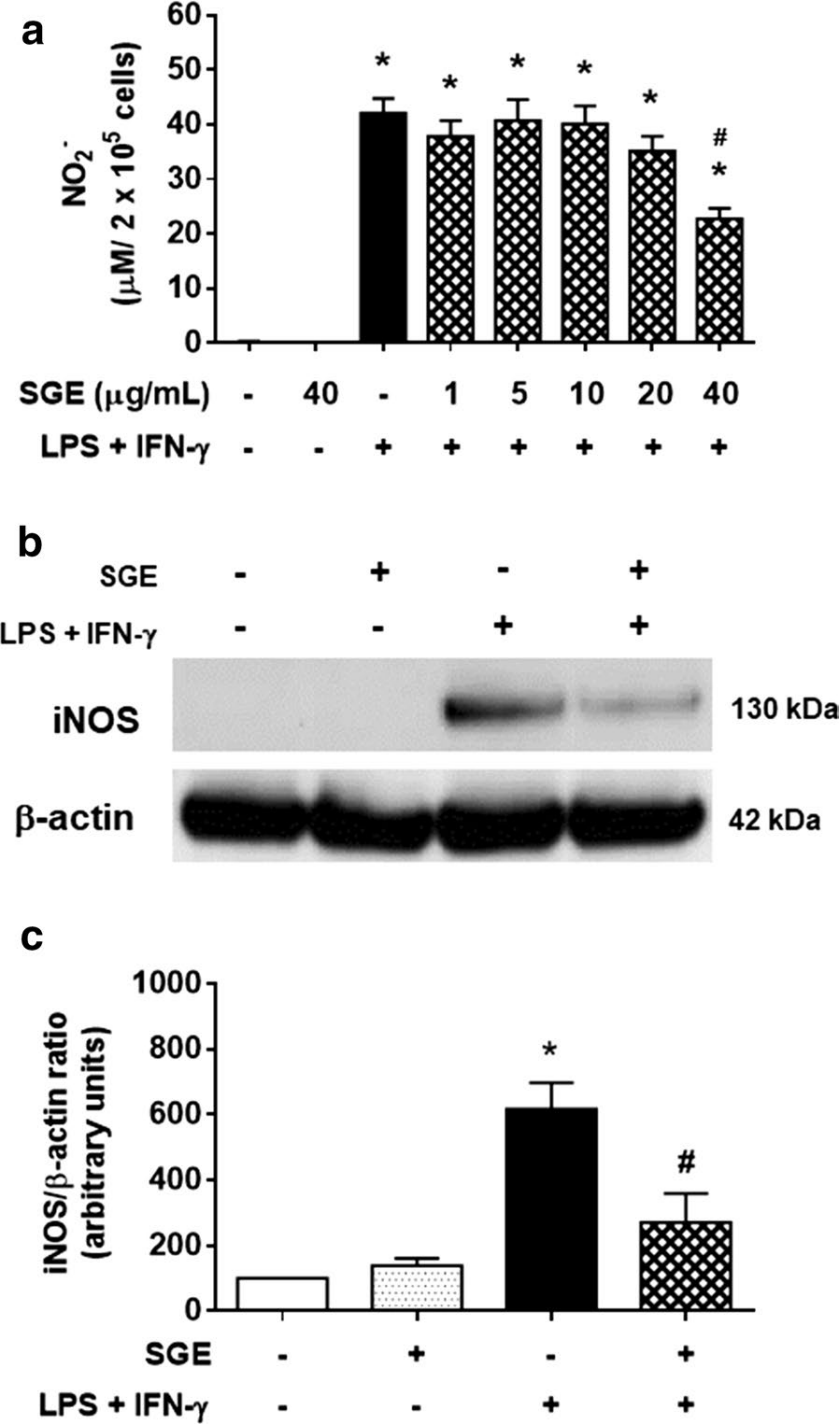
*Aedes aegypti* SGE inhibits NO production and iNOS expression by M1-polarized murine macrophages. Thioglycolate-elicited peritoneal macrophages were collected and cultured as described in “Methods”. Cells were preincubated with complete medium (control group) or with SGE (final concentration indicated in the figure; otherwise 40 µg/ml) for 1 h and stimulated or not with LPS plus IFN-γ (final concentration: 10 ng/ml of each). NO production was indirectly estimated after 48 h in culture supernatants by Griess reaction (**a**). iNOS expression was evaluated after 24 h in cell lysates by Western blot (**b**). The relative expression of iNOS was determined by densitometry and the results were presented as percentage in relation to the control group (considered as 100%) (**c**). Results are expressed as the mean ± SEM. *P < 0.05 *versus* control group (cells incubated with medium only); ^#^*P* < 0.05 *versus* “LPS + IFN-γ” group

Because classically activated (M1) murine macrophages can be characterized by the increased expression of iNOS and production of reactive nitrogen metabolites among other markers [32–34], we first analyzed whether *Ae. aegypti* SGE could affect NO production by LPS-plus IFN-γ-activated macrophages [M(LPS+IFN-γ)]. When maintained in medium only or in the presence of SGE, macrophages produced undetectable amounts of NO, while its production was significantly elevated in M(LPS+IFN-γ) (*F*(7,40) = 48.64, *P *< 0.0001). However, in the presence of SGE, NO levels in M(LPS+IFN-γ) cultures were decreased in a concentration-dependent manner, reaching statistical significance at 40 μg/ml of SGE (*F*(7,40) = 48.64, *P *< 0.0001; Fig. 1a). Due to these initial findings, most of the next assays employed the *Ae. aegypti* SGE at 40 μg/ml concentration. The evaluation of iNOS expression revealed a similar pattern, with the high expression of iNOS in M(LPS+IFN-γ) being reduced in the presence of SGE (Fig. 1b). Densitometry analysis of Western blot bands presented in Fig. 1c revealed that this reduction was statistically significant (*F*(3,8) = 15.04, *P* = 0.0012).

### *Aedes aegypti* SGE induces viability changes in murine lymphocytes, but not in macrophages

**Fig. 2:**
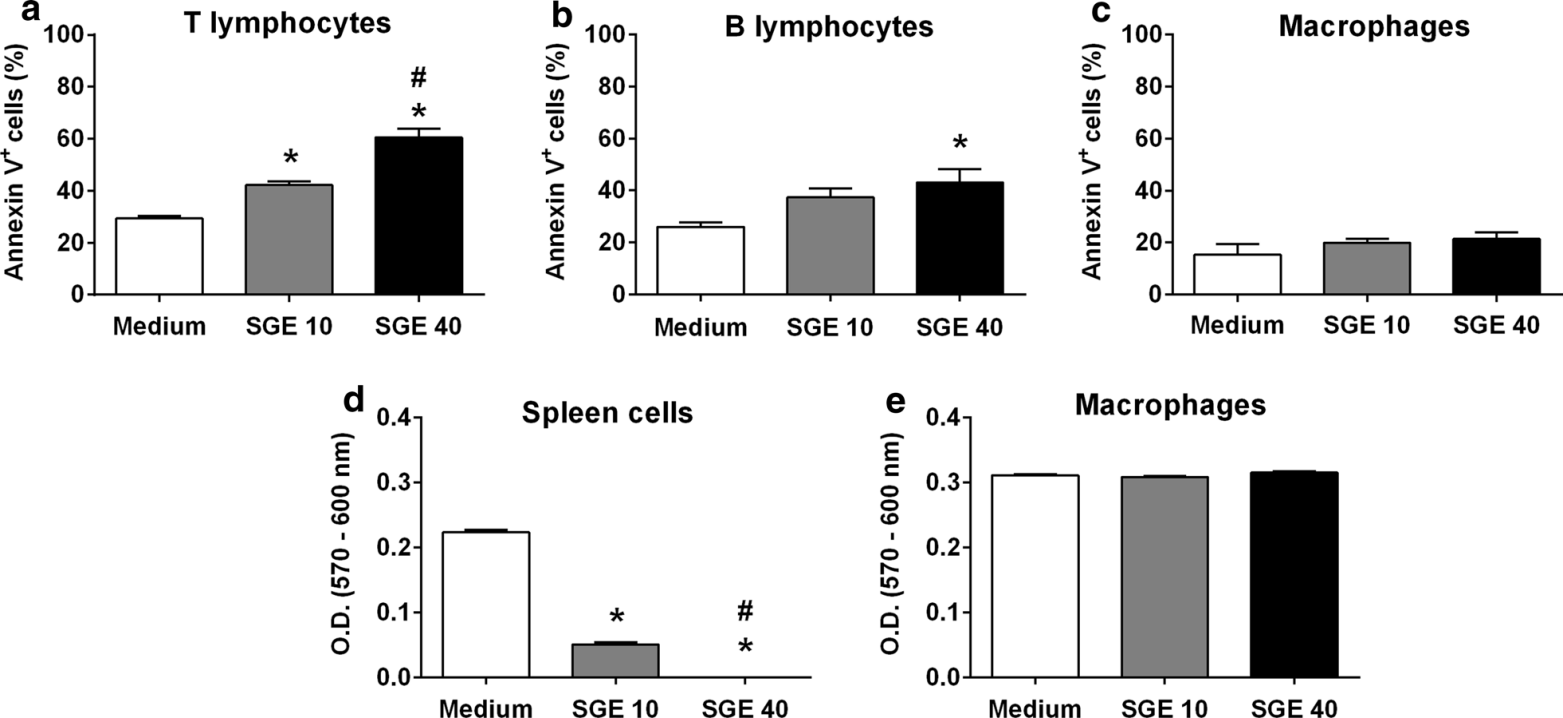
*Aedes aegypti* SGE affects viability of murine lymphocytes but not of macrophages. Thioglycolate-elicited peritoneal macrophages and total spleen cells were collected and cultured as described in “Methods”. Cells were maintained into culture tubes and incubated with complete medium (control group) or with SGE (final concentration: 10 and 40 µg/ml) for 4 h. Annexin V staining was evaluated by flow cytometry in CD3^+^ cells (T lymphocytes) (**a**), CD19^+^ cells (B lymphocytes) (**b**), and CD11b^+^F4/80^+^ cells (macrophages) (**c**). In another set of experiments, cells were preincubated with complete medium (control group) or with SGE (final concentration: 10 and 40 µg/ml) for 1 h and stimulated with Con A (0.5 μg/ml final concentration) for spleen cell cultures (**d**) or LPS plus IFN-γ (10 ng/ml of each, final concentration) for macrophage cultures (**e**). Twenty-five microliters of 0.01% resazurin were added to the cultures and after 48 h, cell viability was evaluated by reading the cultures absorbance at 570 and 600 nm. Results are expressed as the mean ± SEM. *P < 0.05 *versus* control group (cells incubated with medium only); ^#^*P* < 0.05 *versus* “SGE 10” group

Several studies have reported that salivary components of *Ae. aegypti* impair proliferation and induce cell death in lymphocytes [20–22, 35]. In order to determine if *Ae. aegypti* SGE disturbs macrophage viability, the expression of phosphatidylserine on the outer membrane was evaluated in these cells through annexin V staining and compared to T and B lymphocytes. As previously reported [20], annexin V staining is increased in CD3^+^ cells (T lymphocytes, Fig. 2a) and CD19^+^ cells (B lymphocytes, Fig. 2b) in the presence of SGE (*F*(2,9) = 51.55, *P* < 0.0001 and *F*(2,9) = 5.467, *P* = 0.0279, respectively). Of note, significant annexin V staining was observed at 10 μg/ml SGE in T lymphocytes, while the same was observed only at 40 μg/ml SGE in B cells, suggesting that the former is more sensitive than the latter to the SGE-induced cell death. Under the same conditions, the percentage of annexin V^+^ cells was about the same in F4/80^+^/CD11b^+^ cells (macrophages) regardless of the presence of *Ae. aegypti* SGE (*F*(2,8) = 1.033, *P* = 0.3988; Fig. 2c).

To confirm the refractoriness of macrophages to SGE-induced cell death, we performed a viability assay largely used for cells and microorganisms [36–39]. The presence of SGE on spleen cell cultures (largely comprised of lymphocytes) significantly reduced the cell viability in a concentration–dependent manner, starting at SGE concentrations as low as 1 μg/ml (data not shown) and maximal at 40 μg/ml (*F*(2,15) = 1793, *P* < 0.0001; Fig. 2d). On the other hand, when M(LPS+IFN-γ) were incubated under the same conditions, no changes in their viability was observed at any of the SGE concentrations tested (*F*(2,15) = 3.148, *P* = 0.0722; Fig. 2e).

### *Aedes aegypti* SGE decreases spare respiratory capacity in macrophages

**Fig. 3:**
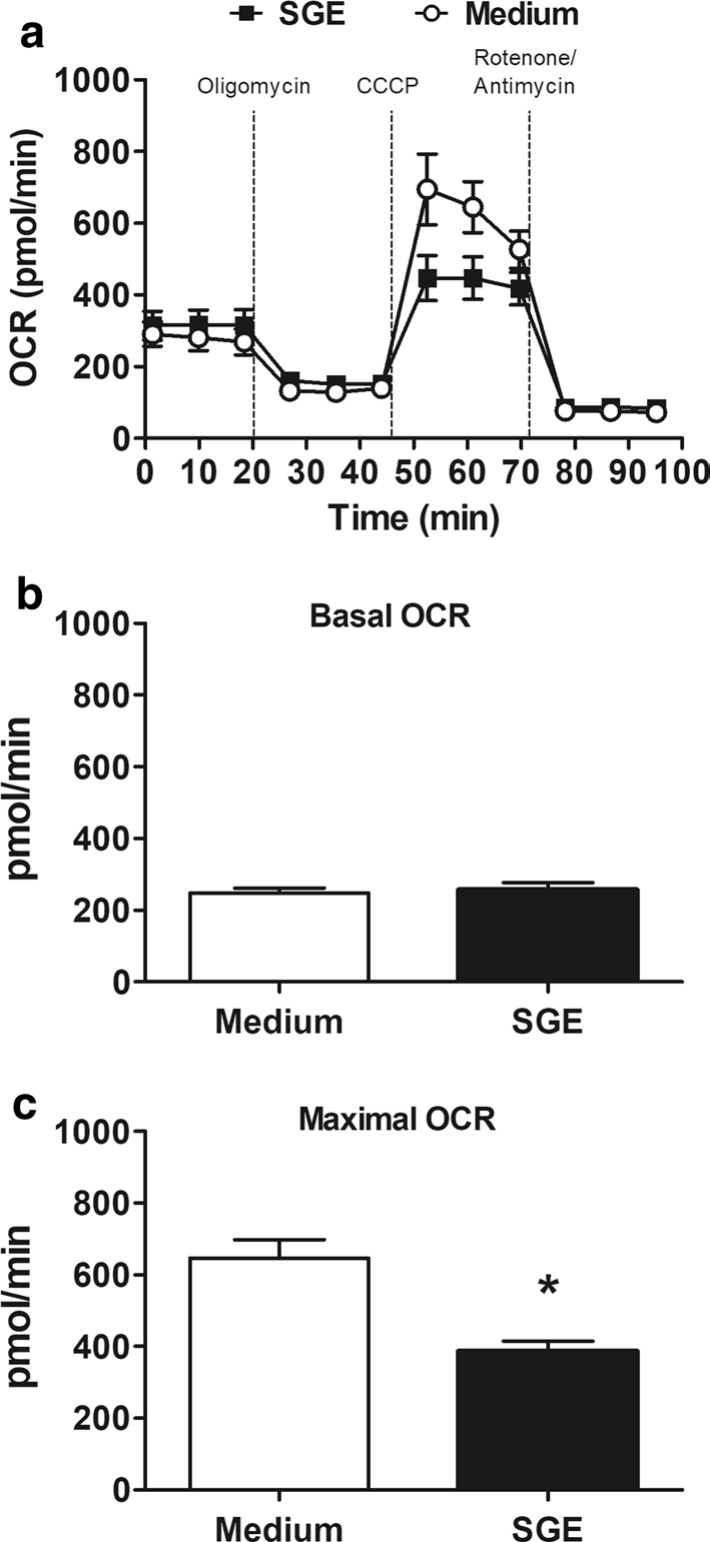
Macrophage spare respiratory capacity is decreased in the presence of *Ae. aegypti* SGE. Thioglycolate-elicited peritoneal macrophages were collected and cultured in Seahorse plates as described in Methods. Mitochondrial function was compared in basal conditions and after the sequential addition of oligomycin (1 μg/ml), CCCP (4.5 μM) and rotenone/antimycin A (1 μM each) on a Seahorse flux analyzer. Basal and maximal OCR are expressed as the mean ± SEM. *P < 0.05 *versus* control group (cells incubated with medium only)

We next evaluated parameters of macrophage mitochondrial respiration in the presence and absence of *Ae. aegypti* SGE as a metabolic parameter of these cells (Fig. 3a). As shown in the Fig. 3b, the basal OCR (energetic demand under baseline conditions) of macrophages incubated with medium or SGE was similar (*t*(4) = 0.3688, *P* = 0.7309). The OCR in the absence of ATP production achieved by oligomycin addition was also similar between the groups. On the other hand, the maximal OCR triggered by CCCP was lower in macrophages incubated with SGE (*t*(4) = 4.407, *P* = 0.0116; Fig. 3c), meaning that the spare respiratory capacity of macrophages (the difference between the basal and the maximal OCR) was decreased in the presence of *Ae. aegypti* SGE.

### Cytokine production by classically activated macrophages (M1) is selectively modulated by *Ae. aegypti* SGE

**Fig. 4:**
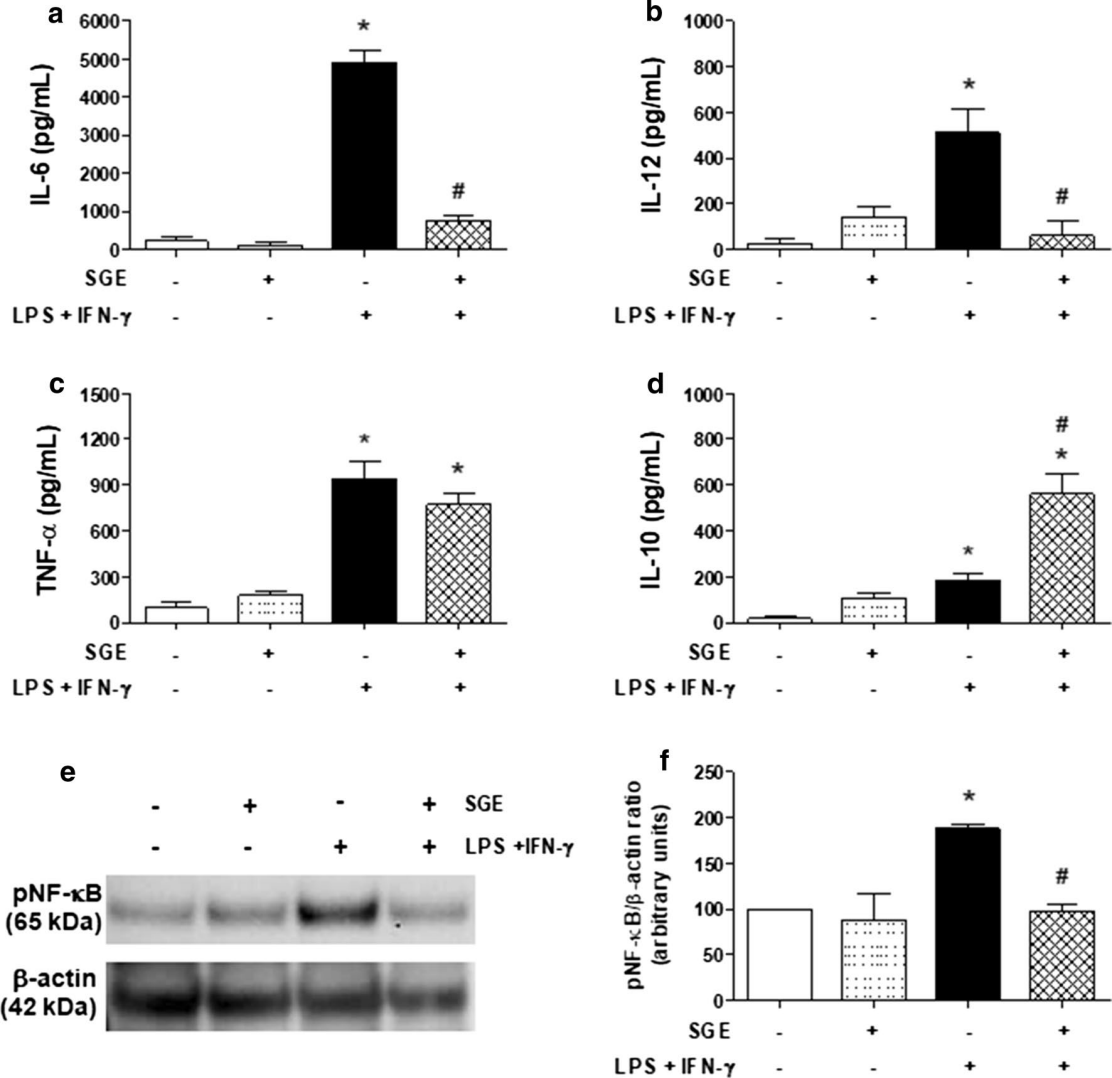
*Aedes aegypti* SGE differentially modulates inflammatory and anti-inflammatory cytokine production, as well as NF-κB expression, by M1-polarized murine macrophages. Thioglycolate-elicited peritoneal macrophages were collected and cultured as described in “Methods”. Cells were preincubated with complete medium (control group) or with SGE (final concentration: 40 µg/ml) for 1 h and then stimulated or not with LPS plus IFN-γ (final concentration: 10 ng/ml of each). Cell culture supernatants were collected after 6 h of culture for TNF-α (**c**) or after 48 h for IL-6 (**a**), IL-12 (**b**) and IL-10 (**d**) determinations by ELISA. Phosphorylated NF-κB (pNF-κB) expression was evaluated after 30 min in cell lysates by Western blot (**e**). The relative expression of pNF-κB was determined by densitometry and the results were presented as percentage in relation to the control group (considered as 100%) (**f**). Results are expressed as the mean ± SEM. *P < 0.05 *versus* control group (cells incubated with medium only); ^#^*P* < 0.05 *versus* “LPS + IFN-γ” group

We next evaluated whether *Ae. aegypti* SGE could also affect the pattern of cytokines induced by classical activation of macrophages. Figure 4 shows that macrophages maintained in medium or in the presence of SGE produced low basal levels of all cytokines evaluated, while M(LPS+IFN-γ) secreted significant levels of IL-6 (*F*(3,12) = 153.8, *P* < 0.0001; Fig. 4a), IL-12 (*F*(3,10) = 9.831, *P* = 0.0025; Fig. 4b), TNF-α (*F*(3,8) = 38.31, *P* < 0.0001; Fig. 4c) and IL-10 (*F*(3,11) = 30.84, *P* < 0.0001; Fig. 4d). Interestingly, the presence of SGE in cultures of M(LPS+IFN-γ) modulated each cytokine in a selective and differential way: whereas IL-6 and IL-12 levels were significantly reduced (Fig. 4a and b, respectively), TNF-α levels were unchanged (Fig. 3c), and IL-10 levels were increased (Fig. 4d).

We next evaluated the expression of phosphorylated NF-κB in these cells to better characterize the mechanisms leading to this modulatory response. Western blot analysis revealed a constitutive expression of this transcriptional factor in macrophages maintained in medium only or in SGE-containing medium. For M(LPS+IFN-γ) samples, augmented expression of phosphorylated NF-κB was observed, while the presence of SGE in the culture reduced its expression to control levels (Fig. 4d). Densitometry analysis of Western blot data showed that this inhibition was statistically significant (*F*(3,4) = 9.776, *P* = 0.0259; Fig. 4e).

### *Aedes aegypti* salivary components do not bind IL-6 or IL-12

**Fig. 5:**
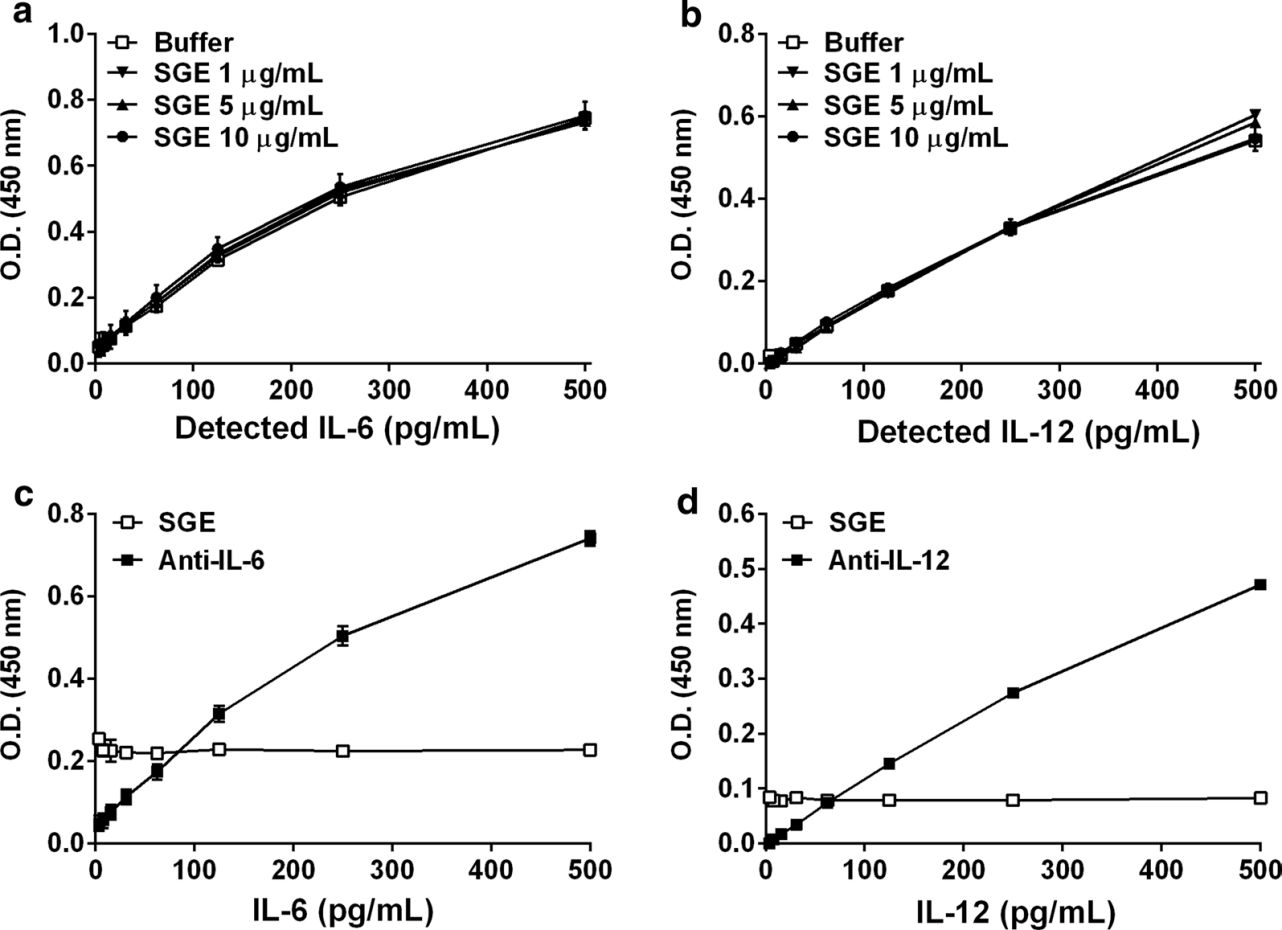
*Aedes aegypti* SGE does not bind murine IL-6 or IL-12. Different concentrations of *Ae. aegypti* SGE (1, 5 and 10 µg/ml) were preincubated with a serial dilution of mouse recombinant IL-6 or IL-12 for 15 minutes at 37 °C. Samples were then transferred to ELISA plates coated with anti-IL-6 or anti-IL-12 capture monoclonal antibody. For each case, a control group (a serial dilution of recombinant cytokine diluted in buffer only) was assayed under the same conditions (**a**, **c**). ELISA plate wells were coated with *Ae. aegypti* SGE (10 µg/ml), anti-IL-6 or anti-IL-12 antibody and incubated with serial dilutions of recombinant IL-6 or IL-12 (**b**, **d**). The detection of the cytokines was performed as described in “Methods”. Results are expressed as the mean ± SEM

In all sets of experiments performed to evaluate the cytokine production by M(LPS+IFN-γ), a consistent decrease in IL-6 and IL-12 detection was achieved when the SGE was present in the culture. Because saliva of many tick species has been demonstrated to present cytokine/chemokine binding proteins [40–43], we investigated whether *Ae. aegypti* would have molecule(s) with similar properties. For competition assays, increasing amounts of SGE were coincubated with standard curves of murine IL-6 or IL-12 before being transferred to the wells coated with the respective capture antibodies. No changes in the detection of IL-6 or IL-12 was observed in the presence of *Ae. aegypti* SGE (Fig. 5a and b, respectively). Likewise, wells coated with SGE did not interact with IL-6 or IL-12 under our experimental conditions in direct binding assays (Fig. 5c and d, respectively).

### M1, but not to M2 polarization, is impaired by *Ae. aegypti* SGE

**Fig. 6:**
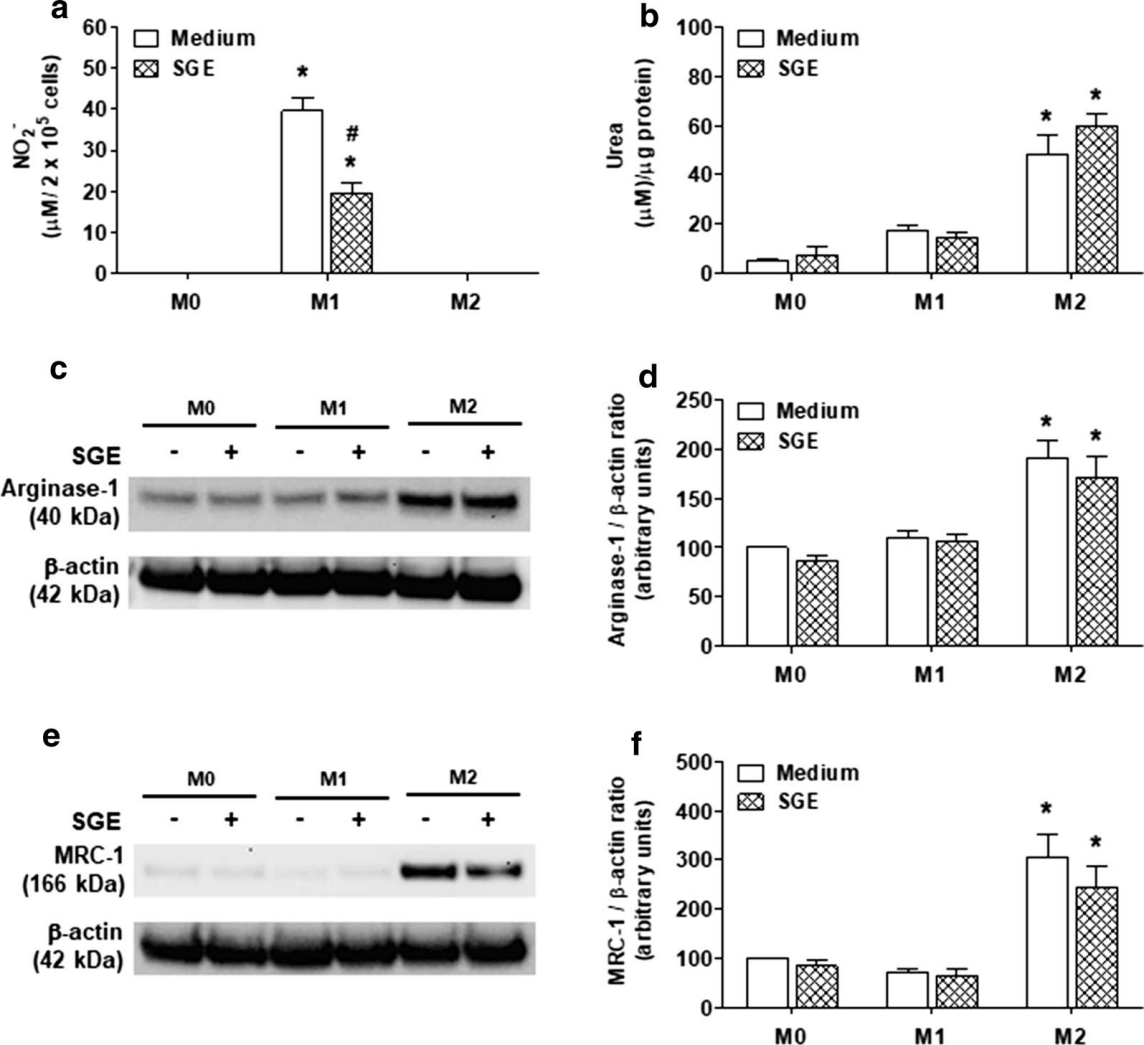
*Aedes aegypti* SGE impairs M1 but not M2 polarization. Thioglycolate-elicited peritoneal macrophages were collected and cultured as described in “Methods”. Cells were preincubated with complete medium (control group) or with SGE (final concentration: 40 µg/ml) for 1 h and maintained in medium (M0 condition), stimulated with LPS plus IFN-γ (final concentration: 10 ng/ml of each) for M1 polarization or with IL-4 (final concentration: 20 ng/ml) for M2 polarization. After 48 h, culture supernatants were collected for NO determination by Griess reaction (**a**) and the cell lysate were prepared for urea determination as a product of the arginase activity (**b**). Arginase-1 (**c**) and mannose receptor-1 (MRC-1) (**e**) expression were evaluated by Western blot. The relative expression of arginase-1 and MRC-1 were determined by densitometry and the results were presented as percentage in relation to the control group (considered as 100%) (**d** and **f**, respectively). Results are expressed as the mean ± SEM. *P < 0.05 *versus* respective control group (cells incubated with medium or SGE only)

Because *Ae. aegypti* salivary components downmodulated some microbicidal and inflammatory mediators typically produced by M1 macrophages, we next asked whether the mosquito SGE could directly induce M2 polarization, or influence M2 polarization induced by IL-4 [M(IL-4)]. Considering the nearly reciprocal patterns of l-arginine metabolism by iNOS and arginase 1 in M1 and M2 macrophages [44, 45], the production of NO and urea, respectively, were used as initial parameters to characterize these subsets. Figure 6a shows that incubation with medium or SGE alone (M0 condition) was not able to induce NO production, as already presented in Fig. 1. Likewise, high levels of NO are produced by M(LPS+IFN-γ), and this production is reduced in the presence of SGE (*F*(5,18) = 93.97, *P* < 0.0001). On the other hand, M(IL-4) did not produce detectable NO either in presence or absence of SGE (Fig. 6a). For M2-associated markers, constitutive basal levels of urea production (Fig. 6b), as well as expression of arginase-1 (Fig. 6c, d) and mannose receptor-1 (Fig. 6e, f), were detected in macrophages incubated with medium or SGE only. Under M1 polarizing conditions, all these markers were equally detected at basal levels, while under M2 polarizing conditions they were all significantly increased (*F*(5,18) = 29.71, *P* < 0.0001 for urea; *F*(5,18) = 11.82, *P* < 0.0001 for arginase-1; *F*(5,24) = 14.65, *P* < 0.0001 for mannose receptor-1). The presence of SGE in both situations did not change urea production or arginase-1/mannose receptor-1 expression.

### *Aedes aegypti* SGE suppresses bacterial internalization and microbicidal activity by macrophages

**Fig. 7:**
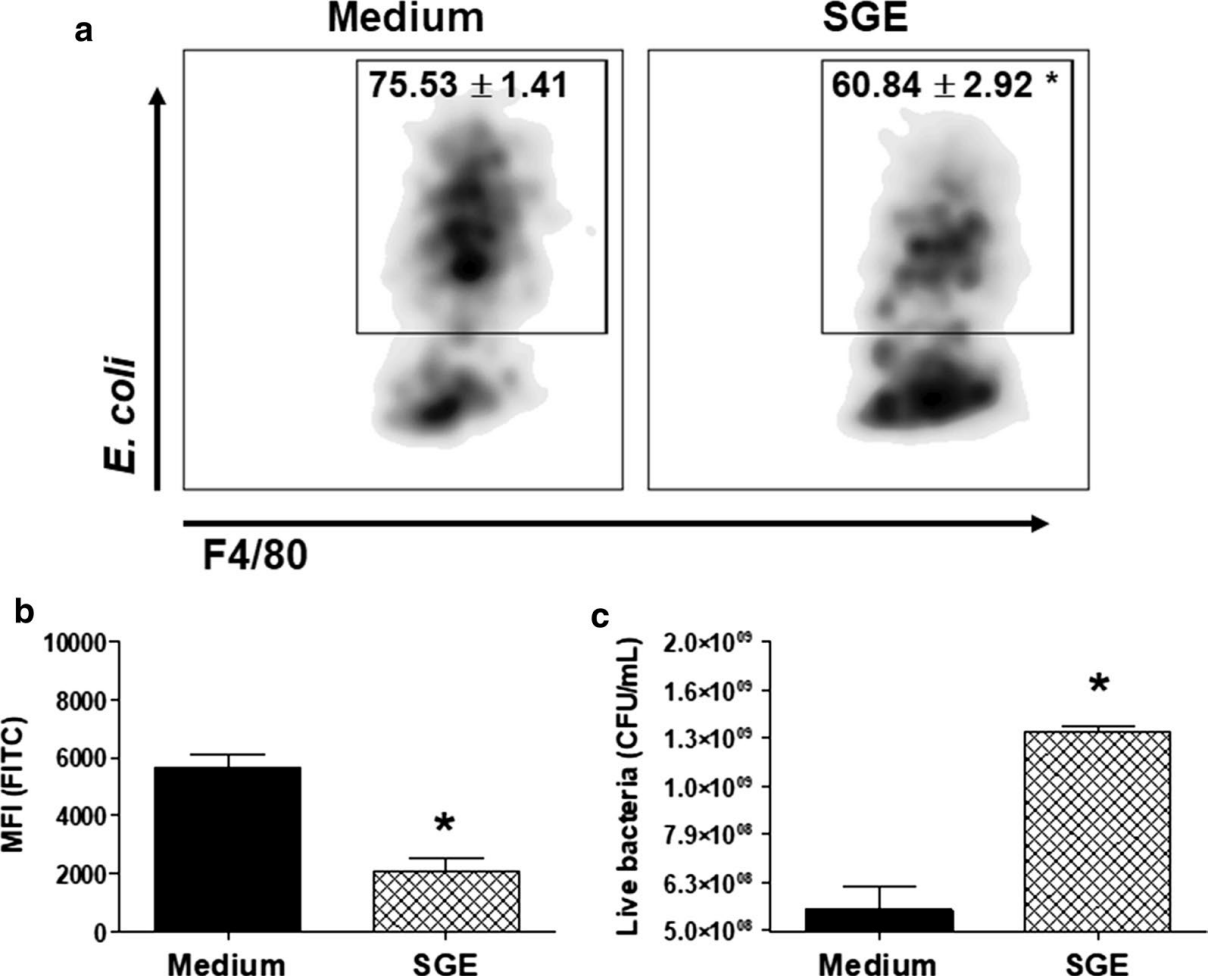
*Aedes aegypti* SGE impairs bacterial internalization and microbicidal activity by murine macrophages. Thioglycolate-elicited peritoneal macrophages were collected and cultured as described in “Methods”. Cells were preincubated with complete medium (control group) or with SGE (final concentration: 40 μg/ml) for 1 h and stimulated with green fluorescent *E. coli* at a multiplicity of infection of 10. Bacterial uptake was evaluated after 1 h by flow cytometry and represented as density plots (**a**) and as median fluorescence intensity (MFI) (**b**) in F4/80^+^ cells (macrophages); the bacterial killing was estimated after 5 h by determination of colony-forming units (**c**). Results are expressed as the mean ± SEM. *P < 0.05 *versus E. coli* group

In addition to the inflammatory mediators produced by classically activated macrophages, their microbicidal role is an ultimate expected phenotype. Thus, we evaluated if the presence of *Ae. aegypti* SGE in the culture would interfere with *E. coli* internalization and killing by macrophages. Fluorescent *E. coli* were observed to be associated to macrophages and preincubation with SGE decreased the percentage of positive cells harboring bacteria (*t*(14) = 4.526, *P* = 0.0005; Fig. 7a) and the fluorescence median intensity (*t*(14) = 5.331, *P* < 0.0001; Fig. 7b). In addition, significantly more bacteria were recovered from macrophages preincubated with SGE in comparison to cells maintained in medium only before the bacterial challenge (*t*(14) = 9.978, *P* < 0.0001; Fig. 7c). Together, these findings suggest that *Ae. aegypti* SGE impairs both the internalization and the microbicidal activity of macrophages.

## Discussion

Since the demonstration that macrophages could be “alternatively” activated in the presence of IL-4 [46], several works expanded this universe and different nomenclatures were proposed to reflect the multitude of macrophage phenotypes and functions shaped by environmental cues associated to health and disease states [47–52]. The term “macrophage polarization” is currently used to express the net result of these conditions, although most researchers in the field agree that given the heterogeneity and plasticity of macrophages, efforts are required to standardize the polarization terminology so that immunologists can speak a common language [26]. Aligned with this viewpoint, in the present work, macrophages activated by LPS plus IFN-γ were interchangeably referred as M1 or M(LPS+IFN-γ) while macrophages activated by IL-4 were referred as M2 or M(IL-4).

Our results revealed that the presence of *Ae. aegypti* SGE in the culture reduced iNOS expression and NO production by M(LPS+IFN-γ). In addition, the presence of *Ae. aegypti* SGE in M(LPS+IFN-γ) cultures also decreased IL-6 and IL-12 production while increasing IL-10 production by these cells, and these changes were associated to diminished expression of phosphorylated NF-κB. Similar inhibition of proinflammatory phenotype was observed in murine macrophages infected with West Nile virus or Sindbis virus in the presence of *Ae. aegypti* SGE [17] and in murine macrophages stimulated by LPS in the presence of *Ae. aegypti* salivary cecropins [24]. Interestingly, the downmodulation of iNOS/NO axis in macrophages is also reported for saliva from ticks [53–56], triatomines [57], sandflies [58–62], horseflies [63] and other mosquito species [64], suggesting that the activation of this antimicrobial pathway is a common target for saliva of hematophagous arthropods. Similarly, inflammatory cytokine production and NF-κB signaling were impaired in bone marrow-derived murine macrophages infected by Zika virus in the presence of LTRIN, an *Ae. aegypti* salivary molecule recently identified and characterized [65]. Likewise, this anti-inflammatory phenotype was also found in the presence of salivary components of many blood-feeding arthropods [53, 56, 66–69]. Despite the characterization of cytokine/chemokine binding proteins in tick saliva [40–43], *Ae. aegypti* salivary molecules seem to directly modulate macrophage biology since no binding of SGE to IL-6 or IL-12 was detected, thus discarding experimental artifacts in our assays.

Salivary preparations from some blood-feeding arthropods were shown to induce death of different cell types. For example, *Lutzomyia longipalpis* SGE induces neutrophil apoptosis [70], while *Armigeres subalbatus* SGE does the same to macrophages [64]. Our group has shown that *Ae. aegypti* SGE induces selective death of naive T cells, but not memory T cells, by a caspase-3- and caspase-8-dependent mechanism [20]. Contrary to that observed for T and B cells, the absence of changes in the annexin V binding or in the viability of macrophages incubated in the presence of SGE suggests that cell death is not the mechanism by which the *Ae. aegypti* saliva modulates the M1 phenotype. The reason for this selectivity is not known, but it might represent differences between lymphocyte and macrophage proliferative rates and susceptibility to cell death. Although some studies revealed that macrophage proliferation has an impact on the homeostatic maintenance [71] and in inflammatory conditions [72, 73], these cells are generally depicted as terminally differentiated and usually dye at the end of their life span. On the contrary, lymphocytes undergo extensive proliferation and death during development of immature stages as well as following activation of mature cells. Thus, cell death events are part of lymphocyte life-cycle in order to eliminate cells that display high affinity antigen receptors for self-antigens or cannot respond to antigens, and also during the contraction phase of immune responses [74]. In this way, a recent comprehensive review highlighted the control of apoptosis by the BCL-2 family of proteins and their differential role on promoting or inhibiting apoptosis depending on the stimuli, the tissue/cell type and the proliferative rate of the cells [75]. The contribution of members of the BCL-2 family on SGE-induced lymphocyte death is a topic of interest for future studies.

The fact that *Ae. aegypti* SGE does not affect the viability of macrophages suggests that changes in the NO and proinflammatory cytokine production, as well as the expression of intracellular proteins associated to these mediators, could be due to a modulatory effect on cell metabolism. Thus, the dynamics of mitochondrial OCR in macrophages maintained in medium or incubated with SGE was evaluated and revealed a profile that was similar to that described by other studies [76–78]. No changes were observed in the basal OCR or ATP-independent OCR of macrophages incubated with SGE. However, the maximal OCR and, consequently, the spare respiratory capacity, were both decreased in macrophages incubated with SGE. These divergent metabolic responses suggest that mitochondrial respiration of macrophages in the presence of *Ae. aegypti* salivary molecules may be associated to the inhibition of M1 inflammatory mediators.

Considering the markers involved in either M1 or M2 polarization, we evaluated whether the presence of SGE in the culture could interfere in the polarization to each phenotype. M1 macrophages metabolize l-arginine to NO through the iNOS while M2 macrophages upregulate arginase-1 levels that shift the l-arginine metabolism to l-ornithine, having urea as the final product, in addition to upregulating mannose receptor. Indeed, we confirmed the NO production by M(LPS+IFN-γ) but not M(IL-4) or resting macrophages, as well as the increased urea production and arginase-1 expression by M(IL-4), when compared to resting macrophages or M(LPS+IFN-γ). Interestingly, the presence of *Ae. aegypti* SGE in the cultures decreased NO production by M(LPS+IFN-γ) but did not alter urea production or arginase-1 and mannose receptor-1 expression by these cells. However, we still cannot rule out an indirect effect of *Ae. aegypti* salivary components on M2 polarization *in vivo*. It is known that *Ae. aegypti* bites induce IL-4 expression at the skin site and the mosquito saliva has a molecule called SAAG-4, capable of programming CD4^+^ T cells to express IL-4 [79]. Our group also demonstrated that the exposure of *Ae. aegypti* bites followed by intranasal challenge with SGE was able to induce the production of IL-4, IL-5 and IL-13 in the lung environment associated to high IgE levels, eosinophil migration and mucus production, evidencing the development of a local Th2 response [80]. Taken together, our data suggests that *Ae. aegypti* SGE interferes in the polarization of M1 macrophages, without affecting the polarization to M2 phenotype *in vitro*, but other parameters need to be further investigated to confirm these results *in vivo*.

In addition to their role in the inflammatory process, an effective M1 response is known to possess cytostatic and/or cytotoxic effects against a number of pathogens. Here, we confirmed that *E. coli* uptake and killing by M(LPS+IFN-γ) were both impaired in the presence of *Ae. aegypti* SGE. Among the effector mediators produced by macrophages, NO is the deepest understood, providing protection against viruses, bacteria, fungi, protozoa and helminths [81]. Macrophages are also among the main targets for the arboviruses transmitted by *Ae. aegypti*; thus, it is plausible to hypothesize that the enhancement of viral transmission by salivary components is associated to their ability to divert the development of a full M1 response. In fact, NO presents antiviral activities *in vivo* and *in vitro* by direct and indirect mechanisms in different disease models [82–84]. However, for *Ae. aegypti*-borne arboviruses, the role of NO in limiting viral replication was shown only for dengue virus to date [85–87], but none of these studies employed murine macrophages. Indeed, our attempts to observe a productive infection of peritoneal murine macrophages with dengue virus have failed so far (data not shown). On the other hand, mosquito studies showed that the presence of a NO donor totally blocked the replication of dengue virus in the hemolymph of a susceptible *Ae. aegypti* strain, while an iNOS inhibitor turned a resistant *Anopheles albimanus* strain permissive to the virus replication [88].

Finally, two previous studies presented elegant insights that are crucial to understand the potential biological relevance of our findings on the *Ae. aegypti*-vertebrate host interactions *in vivo*. Marinotti et al. [89] demonstrated that a successful blood meal depletes ~50% of the total protein from the salivary glands of *Ae. aegypti*. In our hands, one salivary gland pair (SGP) from a five- to eight-day-old *Ae. aegypti* adult female corresponds to 2–4 μg total protein (A. Sá-Nunes, personal communication). Considering that the feeding lesion would have an effective volume of 10 μl [90], the local concentration of saliva inoculated might reach 100–200 μg/ml of protein, about 2.5- to 5-fold the highest concentration used in our assays. Likewise, Wasserman et al. [22] presented a theoretical calculation by which the saliva inoculated by one mosquito was assumed to diffuse through a 1-mm spherical radius, affecting a 4.2 μl volume in the skin. Considering the amount of saliva injected and reingested by the mosquito during the blood meal, the authors calculate that one bite would leave 0.3–0.4 salivary gland pair (SGP) equivalent in the skin, resulting in an effective concentration of saliva as high as 30–70 SGP equivalent/ml at the bite site. Thus, the 40 μg/ml concentration used in our experimental conditions would correspond to 10–20 SGP equivalent/ml and therefore, it is also in the physiological range to modulate the macrophages in the microenvironment of the bite site. Whether this immunomodulatory zone created by the inoculated saliva is able to facilitate arbovirus infection *in vivo* nearby the bite site, it remains to be elucidated.

## Conclusions

The present study reinforces the immunomodulatory role of *Ae. aegypti* salivary components on the vertebrate immune cells. Particularly, we report here changes in the pattern of cytokine production, in the expression of effector molecules involved with the activation of these cells, in parameters of cell metabolism and in the microbial uptake/killing by M1-polarized macrophages, with no effects on M2 polarization *in vitro*. These findings open avenues for studies aimed at determining whether macrophage polarization plays any role in the transmission of arbovirus by *Ae. aegypti*.

## Data Availability

Data supporting the conclusions of this article are included within the article. The datasets used and/or analyzed during the present study are available from the corresponding author upon reasonable request.

## Abbreviations

ANOVA: analysis of variance
CD: cluster of differentiation
Con A: concanavalin A
ELISA: enzyme-linked immunosorbent assay
FBS: fetal bovine serum
IFN-γ: interferon-γ
IL: interleukin
iNOS: nitric oxide synthase
LPS: lipopolysaccharide
PBS: phosphate-buffered saline
MAPK: mitogen-activated protein kinases
NF-κB: nuclear factor-κB
NO: nitric oxide
RPMI: Roswell Park Memorial Institute Medium
SEM: standard error of the mean
SGE: salivary gland extract
TBS-T: Tris-buffered saline with Tween
TNF-α: tumor necrosis factor-α
